# Supplementary material for: Comparison of the Drug-Induced Efficacies between Omidenepag Isopropyl, an EP2 Agonist and PGF2α toward TGF-β2-Modulated Human Trabecular Meshwork (HTM) Cells
Source: J Clin Med. 2022 Mar 16;11(6):1652. doi: 10.3390/jcm11061652 (PMC8954773; doi:10.3390/jcm11061652)
Supplement: Supplementary file 1 [file jcm-11-01652-s001.zip › jcm-1584040-supplementary.pdf]

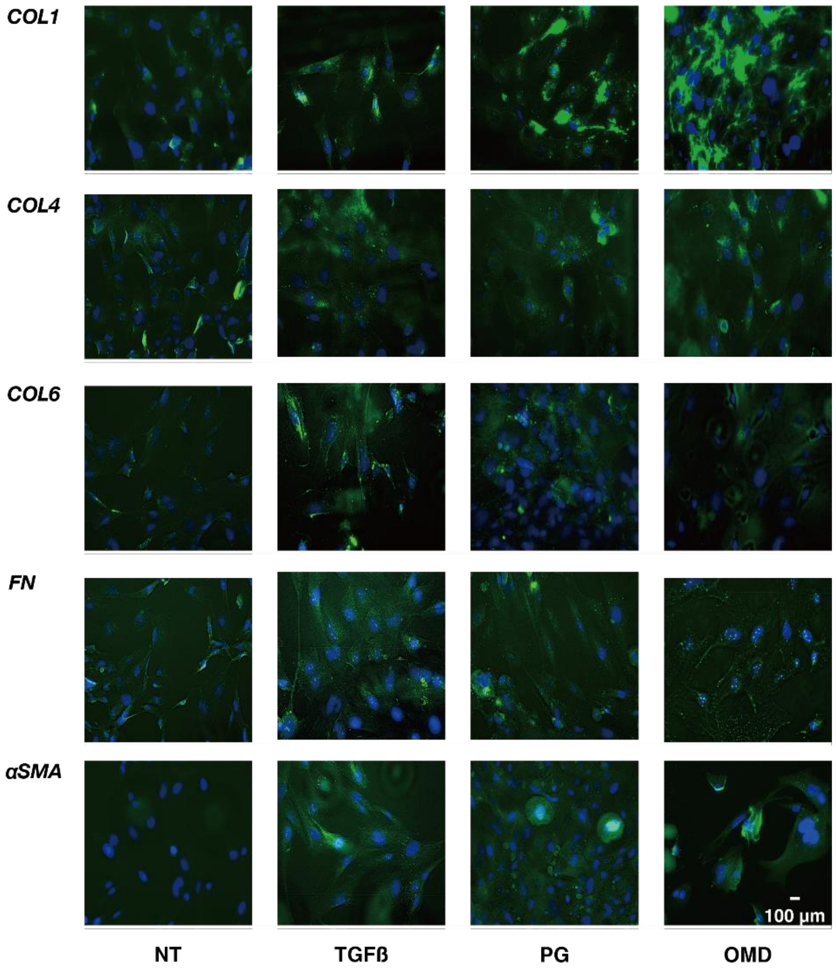

Supplemental Figure S1. Immunofluorescence images of the expressed ECM proteins in 2D cultured HTM cells.

At Day 6, 2D cultured HTM cells (NT: non-treated control) and those treated with a 5 ng/ml solution of TGF-β2 (TGFβ) in the absence and presence of 100 nM PGF2α (PG) or the EP2 agonist, omidenepag (OMD) were immunostained with specific antibodies against ECMs (COL1, COL4, COL6, FN or αSMA; green) and DAPI (blue). Representative immunolabeling images are shown (Scale bar: 100 µm). All experiments were performed in duplicate using fresh preparations (n= 3) and confirmed the consistent immunolabeling among total 6 slides in each experimental condition.

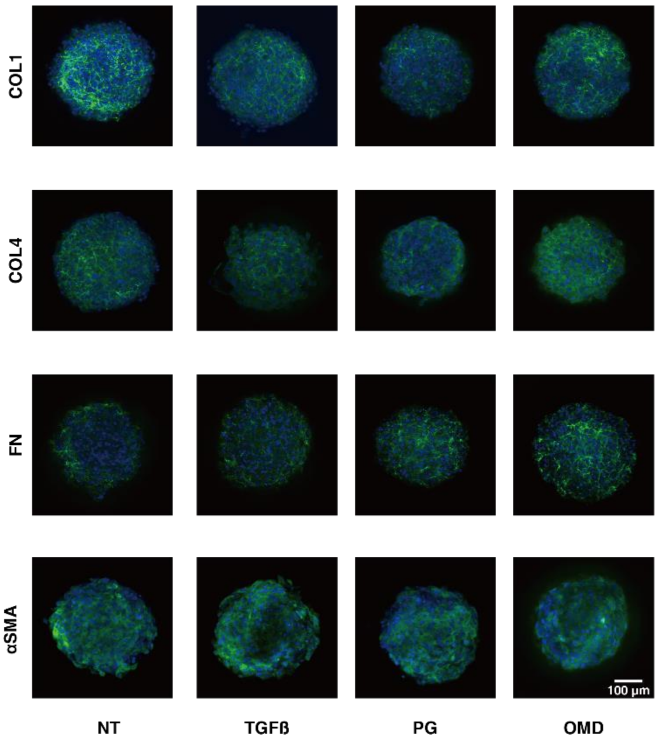

Supplemental Figure S2. Immunofluorescence images of the expressed ECM proteins including COL1, COL4, FN and αSMA in 3D HTM spheroids.

At Day 6, 3D spheroids (NT: non-treated control) and those treated with a 5 ng/ml solution of TGF-β2 (TGFβ) in the absence and presence of 100 nM PGF2α (PG) or the EP2 agonist, omidenepag (OMD) were immunostained with specific antibodies against ECMs (COL1, COL4, FN or αSMA; green) and DAPI (blue). Representative immunolabeling by each antibodies are shown (Scale bar: 100 μm). All experiments were performed in duplicate using fresh preparations consisting of 10 spheroids each.

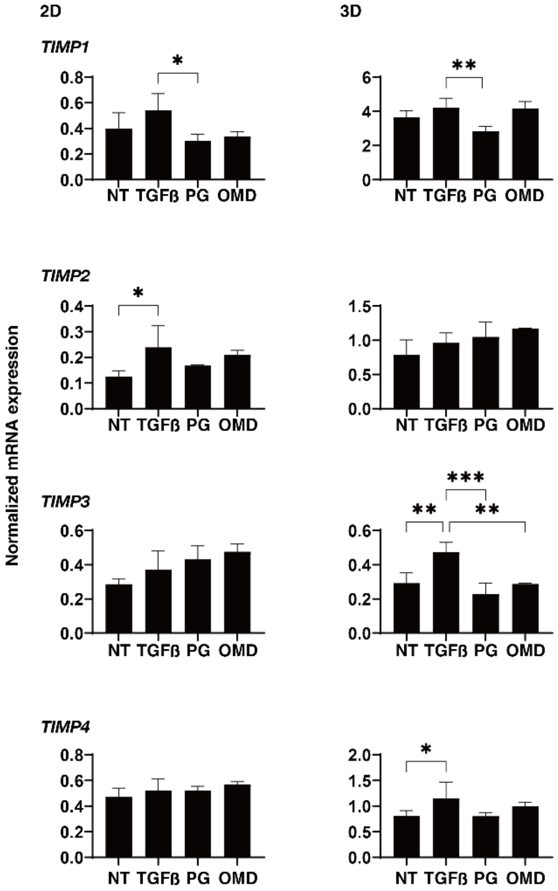

Supplemental Figure S3. Effects of PGF2α and the EP2 agonist, omidenepag (OMD) on the mRNA expression of TIMPs in 2D and 3D cultured HTM cells.

At Day 6, 2D and 3D cultured HTM cells (NT: non-treated control) and those treated with a 5 ng/ml solution of TGF-β2 (TGFβ) in the absence and presence of 100 nM PGF2α (PG) or the EP2 agonist, omidenepag (OMD) were subjected to mRNA expression analysis of mRNA in *TIMP1-4*. Analyses were performed in triplicate using fresh preparations (n=12-15 3D spheroids each). Data presented are the arithmetic mean ± standard error of the mean (SEM), and statistical differences were determined by ANOVA followed by a Tukey's multiple comparison test. **P*<0.05, ***P*<0.01, ****P*<0.005.

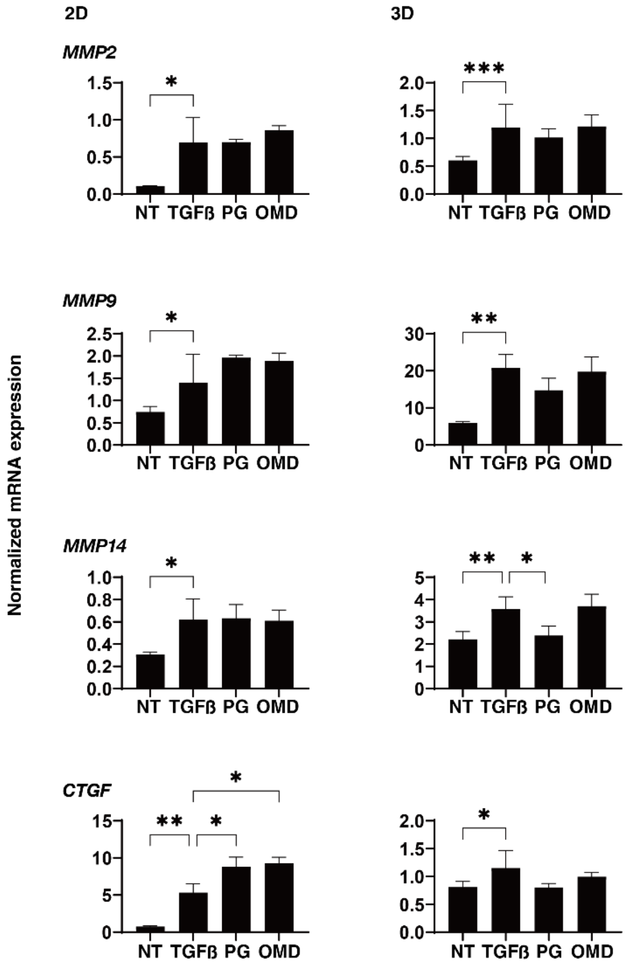

Supplemental Figure S4. Effects of PGF2α and the EP2 agonist, omidenepag (OMD) on the mRNA expression of MMPs and CTGF in 2D and 3D cultured HTM cells.

At Day 6, 2D and 3D cultured HTM cells (NT: non-treated control) and those treated with a 5 ng/ml solution of TGF-β2 (TGFβ) in the absence and presence of 100 nM PGF2α (PG) or the EP2 agonist, omidenepag (OMD) were subjected to mRNA expression analysis of *MMP 2, 9* and *14* and *CTGF*. Analyses. All experiments were performed in duplicate using fresh preparations (n=12-15 3D spheroids each). Data presented are the arithmetic mean ± standard error of the mean (SEM), and statistical differences were determined by ANOVA followed by a Tukey's multiple comparison test. $*P<0.05$, $**P<0.01$, $***P<0.005$.

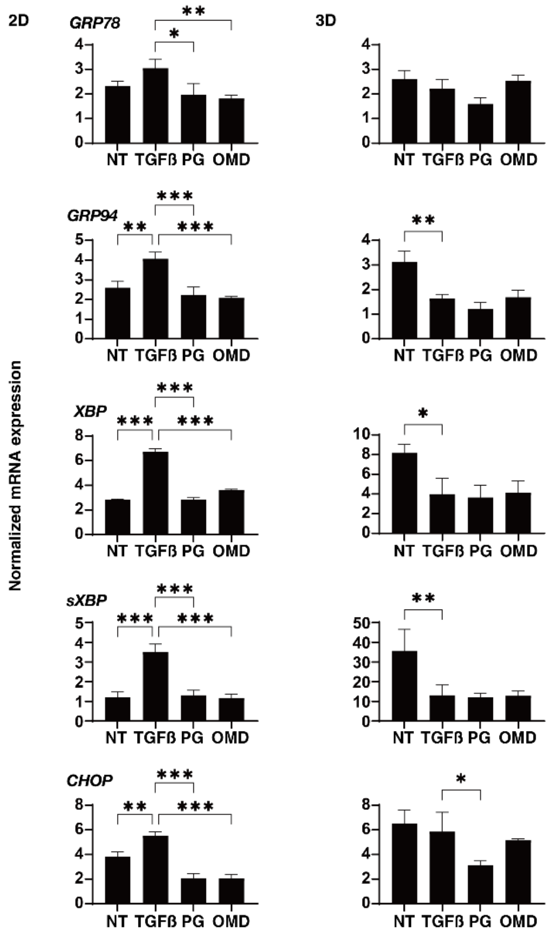

Supplemental Figure S5. Effects of PGF2α and the EP2 agonist, omidenepag (OMD) on the mRNA expression of ER stress related factors in 2D and 3D cultured HTM cells.

At Day 6, 2D and 3D cultured HTM cells (NT: non-treated control) and those treated with a 5 ng/ml solution of TGF-β2 (TGFβ) in the absence and presence of 100 nM PGF2α (PG) or the EP2 agonist, omidenepag (OMD) were subjected to mRNA expression analysis of *GRP78, GRP94, XBP, sXBP* and *CHOP*. Analyses were performed in duplicate using fresh preparations (n=12-15 3D spheroids each). Data presented are the arithmetic mean ± standard error of the mean (SEM), and statistical differences were determined by ANOVA followed by a Tukey's multiple comparison test. **P*<0.05, ***P*<0.01, ****P*<0.005.

Supplemental Table S1A. Sequences of primers used in the qPCR

		Sequence	Exon Location	RefSeq Number	Product Length (bp)
human RPLP0*1	Probe	5'-/56-FAM/CCCTGTCTT/ZEN/CCCTGGGCATCAC/3IABkFQ/-3'	2-3	NM_001002	4413
	Primer2	5'-TCGTCTTTAAACCCTGCGTG-3'			
	Primer1	5'-TGTCTGCTCCCACAATGAAAC-3'			
human COL1A1 *1	Probe	5'-/56-FAM/TCGAGGGCC/ZEN/AAGACGAAGACATC/3IABkFQ/-3'	1-2	NM_000088	17554
	Primer2	5'-GACATGTTCAGCTTTGTGGAC-3'			
	Primer1	5'-TTCTGTACGCAGGTGATTGG-3'			
human COL4A1 *1	Probe	5′-/56-FAM/TCATACAGA/ZEN/CTTGGCAGCGGCT/3IABkFQ/-3′	51-52	NM_001845	158195
	Primer2	5′-AGAGAGGAGCGAGATGTTCA-3′			
	Primer1	5′-TGAGTCAGGCTTCATTATGTTCT-3′			
human COL6A1 *2	Primer2	5'-CCTCGTGGACAAAGTCAAGT-3'	2-3	NM_001848	23279
	Primer1	5'-GTGAGGCCTTGGATGATCTC-3'			
human FN1	Primer2	5'-CGTCCTAAAGACTCCATGATCTG-3'	3-4	NM_212482	75204
	Primer1	5'-ACCAATCTTGTAGGACTGACC-3'			
human αSMA*1	Probe	5'-/56-FAM/AGACCCTGT/ZEN/TCCAGCCATCCTTC/3IABkFQ/-3'	8-9	NM_001613	56324
	Primer2	5'-AGAGTTACGAGTTGCCTGATG-3'			
	Primer1	5'-CTGTTGTAGGTGGTTTCATGGA-3'			
human TIMP1*1	Probe	5′-/56-FAM/TCAACCAGA/ZEN/CCACCTTATACCAGCG/3IABkFQ/-3′	2-4	NM_003254	4382
	Primer2	5′-CCTTCTGCAATTCCGACCT-3′			
	Primer1	5′-GCTTGGAACCCTTTATACATCTTG-3′			
human TIMP2*1	Probe	5′-/56-FAM/TCTCATTGC/ZEN/AGGAAAGGCCGAGG/3IABkFQ/-3′	3-4	NM_003255	72411
	Primer2	5′-GACGTTGGAGGAAAGAAGGA-3′			
	Primer1	5′-TGTGGTTCAGGCTCTTCTTC-3′			
human TIMP3*1	Probe	5′-/56-FAM/CCTCCTTTA/ZEN/CCAGCTTCTTCCCCAC/3IABkFQ/-3′	1-3	NM_000362	61337
	Primer2	5′-CCTTCTGCAACTCCGACATC-3′			
	Primer1	5′-CGGTACATCTTCATCTGCTTGA-3′			
human TIMP4*1	Probe	5′-/56-FAM/ACTGAGGAC/ZEN/CTGACCAGTCAAGAGA/3IABkFQ/-3′	3-4	NM_003256	5845
	Primer2	5′-GGTTTGAGAAAGTCAAGGATGTTC-3′			
	Primer1	5′-GTTGCACAGATGGATGAAGAC-3′			
human MMP2*2	Primer2	5′-TCCACCACCTACAACTTTGAG-3′	6-7	NM_004530	27862
	Primer1	5′-GTGCAGCTGTCATAGGATGT-3′			
human MMP9**2	Primer2	5′-ACATCGTCATCCAGTTTGGTG-3′	3-4	NM_004994	7654
	Primer1	5′-CGTCGAAATGGGCGTCT-3′			
human MMP14*2	Primer2	5′-TTCGCCGACTAAGCAGAAG-3′	1-1	NM_004995	11174
	Primer1	5′-CTTGAATTCCTAGACCGCTGT-3′			
human CTGF*2	Primer2	5′-GAAGCTGACCTGGAAGAGAAC-3′	4-5	NM_001901	3197
	Primer1	5′-GCTCGGTATGTCTTCATGCTG-3′			
human Grp78*2	Forward	5'-CATCACGCCGTCCTATGTCG-3'		NM_005347	6491
	Reverse	5'-CGTCAAAGACCGTGTTCTCG-3'			
human GRP94*2	Forward	5'-CTGGGACTGGGAACTTATGAATG-3'		NM_003299	17517
	Reverse	5'-TCCATATTCGTCAAACAGACCAC-3'			
human XBP*2	Forward	5'-AGTAGCAGCTCAGACTGCCA-3'		NM_005080	6010
	Reverse	5'-CCTGGTTCTCAACTACAAGGC-3'			
**human sXBP*2**	**Forward**	**5'-GGTCTGCTGAGTCCGCAGCAGG-3'**		**AB076384**	**6010**
	**Reverse**	**5'-GGGCTTGGTATATATGTGG-3'**			
Human Chop*2	Forward	5'-GGAGAACCAGGAAACGGAAAC-3'		NM_ 004083	3930
	Reverse	5'-TCTCCTTCATGCGCTGCTTT-3'			
Human ZO1*1	Probe	5'--/56-FAM/ACTGAATTA/ZEN/CCTTCACCATGTGCTCCC/3lABkFQ/-3'	24-25	NM_ 175610	269683
	Primer2	5'-CGCGTCTCTCCACATACATTC-3'			
	Primer1	5'-GCTGGCTTATTCTGAGATGGA-3'			
Human Cldn11*1	Probe	5'--/56-FAM/TGACTGCCT/ZEN/GCTTTGTGCTACGT/3lABkFQ/-3'	2-3	NM_ 001185056	15824
	Primer2	5'-CATGGATTTCAGAACCTGCATT-3'			
	Primer1	5'-GGAAGAACAGTCAGCAGCA-3'			

*1 Taqman probes (IDT, Coralville, IA, USA). *2 SYBR probes (IDT, Coralville, IA, USA).

Supplemental Table S1B. Running protocols for a real time RT-PCR of TaqMan based and SYBR based analyses.

Taqman: Cycling Protocol

Step	Cycles	Temperature (°C)	Cycling (min: sec)
Polymerase activation	1	95	3:00
Amplification	35-45		
Denaturation		95	0:05
Annealing/Extension		60	0:30

SYBR: Cycling Protocol

Step	Cycles	Temperature (°C)	Duration (min: sec)
UDG activation	Hold	50	2:00
Dual-Lock™ DNA polymerase	Hold	95	2:00
Denature	40	95	0:01
Anneal/extend	40	60	0:30

SYBR: Melt curve stage

Step	Ramp rate	Temperature (°C)	Time (min: sec)
1	1.6°C/second	95	0:15
2	1.6°C/second	60	1:00
3*1	0.15°C/second	95	0:15

*1 Dissociation

Supplemental Table S2. Product data of 1st antibodies for immunocytochemistry

	Host	Speacies reactivities	Recommended dilutions for IC
anti-COL1	Rabbit pAb	Human, Bovine	1:50 - 1:200
anti-COL4	Rabbit pAb	Human, Bovine	1:50 - 1:200
anti-COL6	Rabbit pAb	Human, Bovine	1:50 - 1:200
anti- FN	Mouse mAb	Human	1:50 - 1:500
anti-αSMA	Rabbit pAb	Human, Bovine, Guinea pig, Dog, Pig,Rat, Mouse, Chickin	1:50 - 1:200

IC; immuno cytochemistry. COL; collagen. FN; fibronectin. αSMA; α smooth muscle actin. pAb; polyclonal antibody. mAb monoclonal antibody.

Supplemental other material. Certificate of analysis data related primary antibodies used for immunocytochemistry

Phone (484) 791-3823
Fax (484) 369-8654

ROCKLAND™
antibodies & assays
www.rockland-inc.com

P.O. Box 5199
Limerick, PA 19468-5199

CERTIFICATE OF ANALYSIS

Anti-Collagen Type I (RABBIT) Antibody - 600-401-103-0.1

Code: 600-401-103-0.1 **Size:** 100 µg **Lot #:** 41475

Product Description: Anti-Collagen Type I (RABBIT) Antibody - 600-401-103-0.1

Concentration: 1.0 mg/mL by UV absorbance at 280 nm

Physical State: Liquid (sterile filtered)

Label: Unconjugated

Host: Rabbit

Gene Name: COL1A1

Species Reactivity: human, bovine

Buffer: 0.02 M Potassium Phosphate, 0.15 M Sodium Chloride, pH 7.2

Stabilizer: None

Preservative: 0.01% (w/v) Sodium Azide

Expiration: Expiration date is one (1) year from date of opening.

Storage Condition: Store vial at 4° C prior to opening. This product is stable at 4° C as an undiluted liquid. Dilute only prior to immediate use. For extended storage, mix with an equal volume of glycerol, aliquot contents and freeze at -20° C or below. Avoid cycles of freezing and thawing.

Synonyms: rabbit anti-collagen type I antibody, Collagen Of Skin Tendon And Bone, Collagen Type 1 antibody, Collagen type I alpha 1 antibody, Collagen alpha-1 (I) chain, Alpha-1 type I collagen, type 1 procollagen alpha 1

Background: Rockland produces highly active antibodies and conjugates to collagens. Collagens are highly conserved throughout evolution and are characterized by an uninterrupted "Glycine-X-Y" triplet repeat that is a necessary part of the triple helical structure. For these reasons, it is often extremely difficult to generate antibodies with specificities to collagens. The development of 'type' specific antibodies is dependent on NON-DENATURED three-dimensional epitopes. Rockland extensively purifies collagens for immunization from human and bovine placenta and cartilage by limited pepsin digestion and selective salt precipitation. This preparation results in a native conformation of the protein. Antibodies are isolated from rabbit antiserum and are extensively cross-adsorbed by immunoaffinity purification to produce 'type' specific antibodies. Greatly diminished reactivity and selectivity of these antibodies will result if denaturing and reducing conditions are used for SDS-PAGE and immunoblotting. Ideal for investigators involved in Cell Biology, Signal Transduction and Stem Cell research.

Application Note: Anti-Collagen antibodies have been used for indirect trapping ELISA for quantitation of antigen in serum using a standard curve, immunoprecipitation, native (non-denaturing, non-dissociating) PAGE, immunohistochemistry, and western blotting for highly sensitive qualitative analysis.

Purity and Specificity: COLLAGEN I Antibody has been prepared by immunoaffinity chromatography using immobilized antigens followed by extensive cross-adsorption against other collagens, human serum proteins and non-collagen extracellular matrix proteins to remove any unwanted specificities. Typically negligible cross reactivity against other types of collagens was detected by ELISA against purified standards. Some class-specific anti-collagens may be specific for three-dimensional epitopes which may result in diminished reactivity with denatured collagen or formalin-fixed, paraffin embedded tissues. This antibody reacts with most mammalian Type I collagens and has negligible cross-reactivity with Type II, III, IV, V or VI collagens. Non-specific cross-reaction of anti-collagen antibodies with other human serum proteins or non-collagen extracellular matrix proteins is negligible.

Assay Dilutions: All assays should be optimized by the user. Recommended dilutions (if any) may be listed below.

ELISA: 1:5,000 - 1:50,000

FLISA: 1:100

Immunohistochemistry: 1:50 - 1:200

Immunoprecipitation: 1:100

Collagen Type I from human and bovine placenta

Immunogen:

Specific Reference

Stefanovic, B, Schnabl, B, Brenner, DA (2002) Inhibition of collagen alpha 1(I) expression by the 5' stem-loop as a molecular decoy. J.Biol. Chem. 277(20):18229-18237.

Hashimoto, N et al. (2004) Bone marrow-derived progenitor cells in pulmonary fibrosis. J. Clin. Invest. 113:243 -252.

Hazra, S et al. (2004) Peroxisome Proliferator-activated Receptor gamma Induces a Phenotypic Switch from Activated to Quiescent Hepatic Stellate Cells. J. Biol. Chem. 279(12):11392–11401.

She, H, Xiong, S, Hazra, S, Tsukamoto, H (2005) Adipogenic transcriptional regulation of hepatic stellate cells. J. Biol. Chem. 280(6):4959-4967.

Mak KM, Kwong AJ, Chu E, Hoo NM. (2011) Hepatic Steatosis, Fibrosis, and Cancer in Elderly Cadavers. Cancer Biology. 5 DEC 2011. DOI: 10.1002/ar.21525.

Crawford, JR., Pilling D, Gomer, RH (2010) Improved serum-free culture conditions for spleen-derived murine fibrocytes. J Immunol Methods. 363(1): 9–20. Published online 2010 October 1. doi: 10.1016/j.jim.2010.09.025.

Yujie Zhang, Branko Stefanovic. (2016) Akt mediated phosphorylation of LARP6; critical step in biosynthesis of type I collagen. Scientific Reports volume 6, Article number: 22597 (2016) doi:10.1038/srep22597.

Disclaimer:

This product is for research use only and is not intended for therapeutic or diagnostic applications. Please contact a technical service representative for more information. All products of animal origin manufactured by Rockland Immunochemicals are derived from starting materials of North American origin. Collection was performed in United States Department of Agriculture (USDA) inspected facilities and all materials have been inspected and certified to be free of disease and suitable for exportation. All properties listed are typical characteristics and are not specifications. All suggestions and data are offered in good faith but without guarantee as conditions and methods of use of our products are beyond our control. All claims must be made within 30 days following the date of delivery. The prospective user must determine the suitability of our materials before adopting them on a commercial scale. Suggested uses of our products are not recommendations to use our products in violation of any patent or as a license under any patent of Rockland Immunochemicals, Inc. If you require a commercial license to use this material and do not have one, then return this material, unopened to: Rockland Inc., P.O. BOX 5199, Limerick, Pennsylvania, USA.

Related Products:

001-001-103	Bovine COLLAGEN Type I - 001-001-103
009-001-103	Human COLLAGEN Type I - 009-001-103
600-401-103-0.1	Anti-Collagen Type I (RABBIT) Antibody - 600-401-103-0.1
600-401-103-0.5	Anti-Collagen Type I (RABBIT) Antibody - 600-401-103-0.5
600-406-103	Anti-Collagen Type I (RABBIT) Antibody Biotin Conjugated - 600-406-103
611-1302	Anti-RABBIT IgG (H&L) (GOAT) Antibody Peroxidase Conjugated - 611-1302
B304	NORMAL GOAT SERUM (NGS) - B304
MB-070	Blocking Buffer for Fluorescent Western Blotting - MB-070
TMBE-100	TMB ELISA PEROXIDASE SUBSTRATE - TMBE-100
TMBE-1000	TMB ELISA PEROXIDASE SUBSTRATE - TMBE-1000

Relevant Links:

GeneID - 1277 http://www.ncbi.nlm.nih.gov/gene/1277

Phone (484) 791-3823
Fax (484) 369-8654

ROCKLAND™
antibodies & assays
www.rockland-inc.com

P.O. Box 5199
Limerick, PA 19468-5199

NCBI - P02452.5 http://www.ncbi.nlm.nih.gov/protein/P02452.5

UniProtKB - P02452 http://www.uniprot.org/uniprot/P02452

Phone (484) 791-3823
Fax (484) 369-8654

ROCKLAND™
antibodies & assays
www.rockland-inc.com

P.O. Box 5199
Limerick, PA 19468-5199

CERTIFICATE OF ANALYSIS

Anti-Collagen Type IV (RABBIT) Antibody - 600-401-106-0.1

Code: 600-401-106-0.1 **Size:** 100 µg **Lot #:** 40639

Product Description: Anti-Collagen Type IV (RABBIT) Antibody - 600-401-106-0.1

Concentration: 1 mg/ml by UV absorbance at 280 nm

Physical State: Liquid (sterile filtered)

Label: Unconjugated

Host: Rabbit

Gene Name: COL4A1

Species Reactivity: human, bovine

Buffer: 0.02 M Potassium Phosphate, 0.15 M Sodium Chloride, pH 7.2

Stabilizer: None

Preservative: 0.01% (w/v) Sodium Azide

Expiration: Expiration date is one (1) year from date of opening.

Storage Condition: Store vial at 4° C prior to opening. This product is stable at 4° C as an undiluted liquid. Dilute only prior to immediate use. For extended storage, mix with an equal volume of glycerol, aliquot contents and freeze at -20° C or below. Avoid cycles of freezing and thawing.

Synonyms: rabbit anti-Collagen Type IV antibody, Arresten antibody, Canstatin antibody, Collagen Of Basement Membrane Alpha 1 Chain antibody, Collagen alpha-1 (IV) chain, COL4A1

Background: Rockland produces highly active antibodies and conjugates to collagens. Collagens are highly conserved throughout evolution and are characterized by an uninterrupted "Glycine-X-Y" triplet repeat that is a necessary part of the triple helical structure. For these reasons, it is often extremely difficult to generate antibodies with specificities to collagens. The development of 'type' specific antibodies is dependent on NON-DENATURED three-dimensional epitopes. Rockland extensively purifies collagens for immunization from human and bovine placenta and cartilage by limited pepsin digestion and selective salt precipitation. This preparation results in a native conformation of the protein. Antibodies are isolated from rabbit antiserum and are extensively cross-adsorbed by immunoaffinity purification to produce 'type' specific antibodies. Greatly diminished reactivity and selectivity of these antibodies will result if denaturing and reducing conditions are used for SDS-PAGE and immunoblotting.

Application Note: Anti-Collagen antibodies have been used for indirect trapping ELISA for quantitation of antigen in serum using a standard curve, immunoprecipitation, native (non-denaturing, non-dissociating) PAGE, immunohistochemistry, and western blotting for highly sensitive qualitative analysis.

Purity and Specificity: Anti-Collagen Type IV has been prepared by immunoaffinity chromatography using immobilized antigens followed by extensive cross-adsorption against other collagens, human serum proteins and non-collagen extracellular matrix proteins to remove any unwanted specificities. Typically negligible cross-reactivity against other types of collagens was detected by ELISA against purified standards. Some class-specific anti-collagens may be specific for three-dimensional epitopes which may result in diminished reactivity with denatured collagen or formalin-fixed, paraffin embedded tissues. This antibody reacts with most mammalian Type IV collagens and has negligible cross-reactivity with Type I, II, III, V or VI collagens. Non-specific cross-reaction of anti-collagen antibodies with other human serum proteins or non-collagen extracellular matrix proteins is negligible.

Assay Dilutions: All assays should be optimized by the user. Recommended dilutions (if any) may be listed below.

ELISA: 1:5,000 - 1:50,000

Immunohistochemistry: 1:50 - 1:200

Immunoprecipitation: 1:100

Western Blot: 1:1,000 - 1:10,000

Collagen Type IV from human and bovine placenta

Immunogen:

Specific Reference

Hibiki Vincent Tanaka, Nathaniel Chuen Yin Ng, Zhan Yang Yu, Martin Miguel Casco-Robles, Fumiaki Maruo, Panagiotis A. Tsonis, Chikafumi Chiba. (2016) A developmentally regulated switch from stem cells to dedifferentiation for limb muscle regeneration in newts. Nature Communications vol 7, Article: 11069. doi:10.1038/ncomms11069.

Disclaimer:

This product is for research use only and is not intended for therapeutic or diagnostic applications. Please contact a technical service representative for more information. All products of animal origin manufactured by Rockland Immunochemicals are derived from starting materials of North American origin. Collection was performed in United States Department of Agriculture (USDA) inspected facilities and all materials have been inspected and certified to be free of disease and suitable for exportation. All properties listed are typical characteristics and are not specifications. All suggestions and data are offered in good faith but without guarantee as conditions and methods of use of our products are beyond our control. All claims must be made within 30 days following the date of delivery. The prospective user must determine the suitability of our materials before adopting them on a commercial scale. Suggested uses of our products are not recommendations to use our products in violation of any patent or as a license under any patent of Rockland Immunochemicals, Inc. If you require a commercial license to use this material and do not have one, then return this material, unopened to: Rockland Inc., P.O. BOX 5199, Limerick, Pennsylvania, USA.

Related Products:

001-001-106	Bovine COLLAGEN Type IV - 001-001-106
009-001-106	Human COLLAGEN Type IV - 009-001-106
600-401-106-0.5	Anti-Collagen Type IV (RABBIT) Antibody - 600-401-106-0.5
600-406-106	Anti-Collagen Type IV (RABBIT) Antibody Biotin Conjugated - 600-406-106
611-1302	Anti-RABBIT IgG (H&L) (GOAT) Antibody Peroxidase Conjugated - 611-1302
B304	NORMAL GOAT SERUM (NGS) - B304
MB-070	Blocking Buffer for Fluorescent Western Blotting - MB-070
TMBE-1000	TMB ELISA PEROXIDASE SUBSTRATE - TMBE-1000

Relevant Links:

GeneID - 1282	http://www.ncbi.nlm.nih.gov/gene/1282
NCBI - P02462.3	http://www.ncbi.nlm.nih.gov/protein/P02462.3
UniProtKB - P02462	http://www.uniprot.org/uniprot/P02462

Phone (484) 791-3823
Fax (484) 369-8654

ROCKLAND™
antibodies & assays
www.rockland-inc.com

P.O. Box 5199
Limerick, PA 19468-5199

CERTIFICATE OF ANALYSIS

Anti-Collagen Type VI (RABBIT) Antibody - 600-401-108-0.1

Code: 600-401-108-0.1 **Size:** 100 µg **Lot #:** 40725

Product Description: Anti-Collagen Type VI (RABBIT) Antibody - 600-401-108-0.1

Concentration: 1 mg/ml

Physical State: Liquid (sterile filtered)

**Label:**	Unconjugated
**Host:**	Rabbit
**Gene Name:**	COL6A1
**Species Reactivity:**	human, bovine
**Buffer:**	0.125 M Sodium Borate, 0.075 M Sodium Chloride, 0.005 M EDTA, pH 8.0
**Stabilizer:**	None
**Preservative:**	0.01% (w/v) Sodium Azide
**Expiration:**	Expiration date is one (1) year from date of opening.
**Storage Condition:**	Store vial at 4° C prior to opening. This product is stable at 4° C as an undiluted liquid. Dilute only prior to immediate use. For extended storage, mix with an equal volume of glycerol, aliquot contents and freeze at -20° C or below. Avoid cycles of freezing and thawing.
**Synonyms:**	rabbit anti-Collagen Type VI antibody, Collagen alpha-1 (VI) chain, Collagen VI antibody, Human mRNA for collagen VI alpha 1 C terminal globular domain antibody
**Background:**	Rockland produces highly active antibodies and conjugates to collagens. Collagens are highly conserved throughout evolution and are characterized by an uninterrupted "Glycine-X-Y" triplet repeat that is a necessary part of the triple helical structure. For these reasons, it is often extremely difficult to generate antibodies with specificities to collagens. The development of 'type' specific antibodies is dependent on NON-DENATURED three-dimensional epitopes. Rockland extensively purifies collagens for immunization from human and bovine placenta and cartilage by limited pepsin digestion and selective salt precipitation. This preparation results in a native conformation of the protein. Antibodies are isolated from rabbit antiserum and are extensively cross-adsorbed by immunoaffinity purification to produce 'type' specific antibodies. Greatly diminished reactivity and selectivity of these antibodies will result if denaturing and reducing conditions are used for SDS-PAGE and immunoblotting.
**Application Note:**	Anti-Collagen antibodies have been used for indirect trapping ELISA for quantitation of antigen in serum using a standard curve, immunoprecipitation, native (non-denaturing, non-dissociating) PAGE, immunohistochemistry, and western blotting for highly sensitive qualitative analysis.
**Purity and Specificity:**	Anti-Collagen Type VI has been prepared by immunoaffinity chromatography using immobilized antigens followed by extensive cross-adsorption against other collagens, human serum proteins and non-collagen extracellular matrix proteins to remove any unwanted specificities. Typically negligible cross reactivity against other types of collagens was detected by ELISA against purified standards. Some class specific anti-collagens may be specific for three-dimensional epitopes which may result in diminished reactivity with denatured collagen or formalin-fixed, paraffin embedded tissues. This antibody reacts with most mammalian Type VI collagens and has negligible cross-reactivity with Type I, II, III, IV or V collagens. Non-specific cross-reaction of anti-collagen antibodies with other human serum proteins or non-collagen extracellular matrix proteins is negligible.
**Assay Dilutions:**	All assays should be optimized by the user. Recommended dilutions (if any) may be listed below.
**Immunogen:**	Collagen Type VI from human and bovine placenta
**Disclaimer:**	This product is for research use only and is not intended for therapeutic or diagnostic applications. Please contact a technical service representative for more information. All products of animal origin manufactured by Rockland Immunochemicals are derived from starting materials of North American origin. Collection was performed in United States Department of Agriculture (USDA) inspected facilities and all materials have been inspected and certified to be free of disease and suitable for exportation. All properties listed are typical

characteristics and are not specifications. All suggestions and data are offered in good faith but without guarantee as conditions and methods of use of our products are beyond our control. All claims must be made within 30 days following the date of delivery. The prospective user must determine the suitability of our materials before adopting them on a commercial scale. Suggested uses of our products are not recommendations to use our products in violation of any patent or as a license under any patent of Rockland Immunochemicals, Inc. If you require a commercial license to use this material and do not have one, then return this material, unopened to: Rockland Inc., P.O. BOX 5199, Limerick, Pennsylvania, USA.

Related Products:

001-001-108	Bovine COLLAGEN Type VI - 001-001-108
009-001-108	Human COLLAGEN Type VI - 009-001-108
600-401-108-0.5	Anti-Collagen Type VI (RABBIT) Antibody - 600-401-108-0.5
600-406-108	Anti-Collagen Type VI (RABBIT) Antibody Biotin Conjugated - 600-406-108
611-1302	Anti-RABBIT IgG (H&L) (GOAT) Antibody Peroxidase Conjugated - 611-1302
B304	NORMAL GOAT SERUM (NGS) - B304
MB-070	Blocking Buffer for Fluorescent Western Blotting - MB-070
TMBE-100	TMB ELISA PEROXIDASE SUBSTRATE - TMBE-100
TMBE-1000	TMB ELISA PEROXIDASE SUBSTRATE - TMBE-1000

Relevant Links:

GeneID - 1291	http://www.ncbi.nlm.nih.gov/gene/1291
NCBI - AAH52575.1	http://www.ncbi.nlm.nih.gov/protein/AAH52575.1
UniProtKB - P12109	http://www.uniprot.org/uniprot/P12109

Fibronectin (EP5): sc-8422

BACKGROUND

Fibronectin is an extracellular matrix glycoprotein present on most cell surfaces, in extracellular fluids and in plasma. A high molecular weight heterodimeric protein, it was originally discovered as a protein missing from the surfaces of virus-transformed cells, and it has been shown to be involved in various functions including cell adhesion, cell motility and wound healing. Alternative splicing and glycosylation give rise to several different forms of Fibronectin, some of which exhibit restricted tissue distribution or association with malignancies. It has been shown that myofibroblast phenotype formation correlates with the occurrence of glycosylated Fibronectin and Fibronectin splice variants in Dupuytren's disease.

CHROMOSOMAL LOCATION

Genetic locus: FN1 (human) mapping to 2q35; Fn1 (mouse) mapping to 1 C3.

SOURCE

Fibronectin (EP5) is a mouse monoclonal antibody raised against a T-cell leukemia biopsy of human origin.

PRODUCT

Each vial contains 200 µg IgG_1 kappa light chain in 1.0 ml of PBS with < 0.1% sodium azide and 0.1% gelatin.

Fibronectin (EP5) is available conjugated to agarose (sc-8422 AC), 500 µg/0.25 ml agarose in 1 ml, for IP; to HRP (sc-8422 HRP), 200 µg/ml, for WB, IHC(P) and ELISA; and to either phycoerythrin (sc-8422 PE), fluorescein (sc-8422 FITC), Alexa Fluor® 488 (sc-8422 AF488) or Alexa Fluor® 647 (sc-8422 AF647), 200 µg/ml, for IF, IHC(P) and FCM.

Alexa Fluor® is a trademark of Molecular Probes, Inc., Oregon, USA

APPLICATIONS

Fibronectin (EP5) is recommended for detection of Fibronectin of mouse, rat and human origin by Western Blotting (starting dilution 1:200, dilution range 1:100-1:1000), immunoprecipitation [1-2 µg per 100-500 µg of total protein (1 ml of cell lysate)], immunofluorescence (starting dilution 1:50, dilution range 1:50-1:500), immunohistochemistry (including paraffin-embedded sections) (starting dilution 1:50, dilution range 1:50-1:500) and solid phase ELISA (starting dilution 1:30, dilution range 1:30-1:3000).

Suitable for use as control antibody for Fibronectin siRNA (h): sc-29315, Fibronectin siRNA (m): sc-35371, Fibronectin shRNA Plasmid (h): sc-29315-SH, Fibronectin shRNA Plasmid (m): sc-35371-SH, Fibronectin shRNA (h) Lentiviral Particles: sc-29315-V and Fibronectin shRNA (m) Lentiviral Particles: sc-35371-V.

Molecular Weight of Fibronectin: 220 kDa.

Positive Controls: Hep G2 cell lysate: sc-2227, CCD-1064Sk cell lysate: sc-2263 or human platelet extract: sc-363773.

STORAGE

Store at 4° C, **DO NOT FREEZE**. Stable for one year from the date of shipment. Non-hazardous. No MSDS required.

RESEARCH USE

For research use only, not for use in diagnostic procedures.

DATA

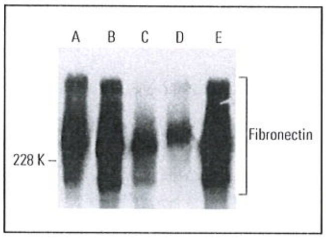

Fibronectin (EP5) HRP: sc-8422 HRP. Direct western blot analysis of Fibronectin expression in Hep G2 (**A**), CCD-1064Sk (**B**), U-87 MG (**C**) and Caki-1 (**D**) whole cell lysates and human platelet extract (**E**).

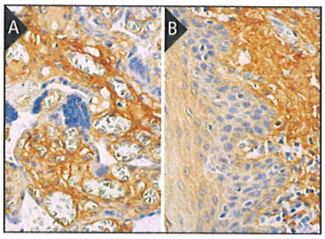

Fibronectin (EP5): sc-8422. Immunoperoxidase staining of formalin fixed, paraffin-embedded human placenta tissue showing staining of extracellular matrix (**A**). Immunoperoxidase staining of formalin fixed, paraffin-embedded human esophagus tissue showing staining of extracellular matrix and cytoplasmic staining of squamous epithelial cells (**B**).

SELECT PRODUCT CITATIONS

1. Hanekamp, E.E., et al. 2003. Consequences of loss of progesterone receptor expression in development of invasive endometrial cancer. Clin. Cancer Res. 9: 4190-4199.
2. Leu, J.I., et al. 2003. Massive hepatic apoptosis associated with TGF-β1 activation after Fas ligand treatment of IGF binding protein-1-deficient mice. J. Clin. Invest. 111: 129-139.
3. Vial, E., et al. 2003. ERK-MAPK signaling coordinately regulates activity of Rac 1 and Rho A for tumor cell motility. Cancer Cell 4: 67-79.
4. Schnabl, B., et al. 2003. Replicative senescence of activated human hepatic stellate cells is accompanied by a pronounced inflammatory but less fibrogenic phenotype. Hepatology 37: 653-664.
5. Wang, G., et al. 2016. STAT3 selectively interacts with Smad3 to antagonize TGF-β. Oncogene 35: 4388-4398.
6. Gezginci-Oktayoglu, S., et al. 2016. Vitamin U has a protective effect on valproic acid-induced renal damage due to its anti-oxidant, anti-inflammatory, and anti-fibrotic properties. Protoplasma 253: 127-135.
7. Kakabadze, Z., et al. 2016. Clinical application of decellularized and lyophilized human amnion/chorion membrane grafts for closing post-laryngectomy pharyngocutaneous fistulas. J. Surg. Oncol. 113: 538-543.
8. Choi, D.H., et al. 2016. Growth factors-loaded stents modified with hyaluronic acid and heparin for induction of rapid and tight re-endothelialization. Colloids Surf. B Biointerfaces 141: 602-610.
9. Hwang, M.P., et al. 2016. Approximating bone ECM: crosslinking directs individual and coupled osteoblast/osteoclast behavior. Biomaterials 103: 22-32.

PROTOCOLS

See our web site at www.scbt.com for detailed protocols and support products.

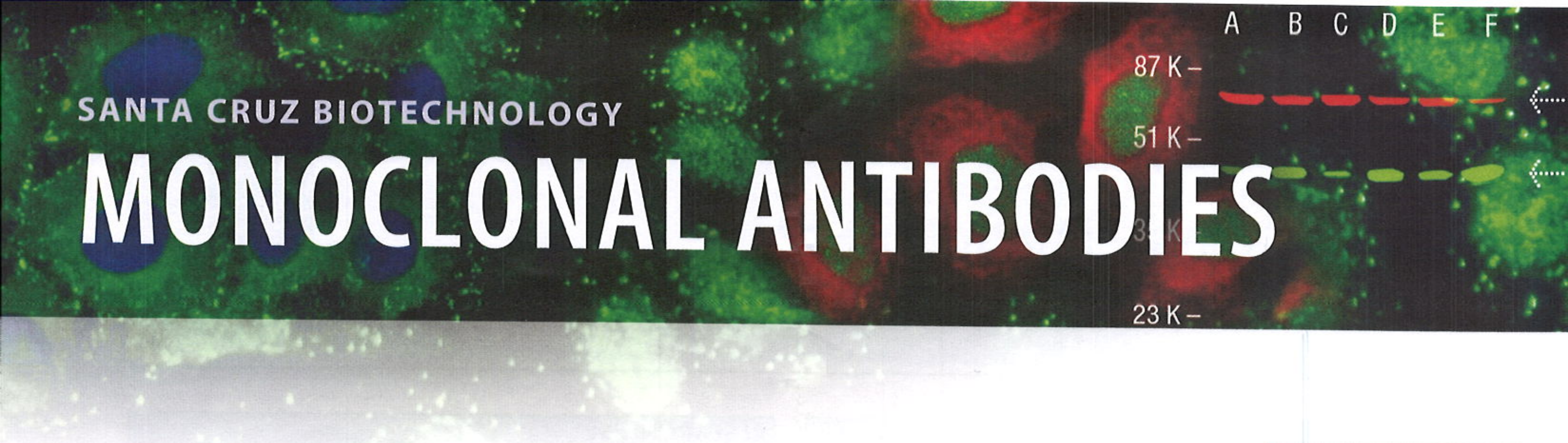

SANTA CRUZ BIOTECHNOLOGY

MONOCLONAL ANTIBODIES

Monoclonal Antibody Advantages

- Strong signal with little non-specific background
- Epitope specificity
- Minimal lot to lot variation
- Uniquely suited for Direct (HRP, FITC, etc.) Conjugates

Mouse IgG Binding Proteins

Achieve superior results using our mouse IgG Binding Proteins.

- Alternatives to conventional secondary antibodies
- Highly specific & selective reagents
- Strong signal with minimal background
- IgG Binding Proteins include HRP, FITC, PE, Biotin & CruzFluor™ conjugates

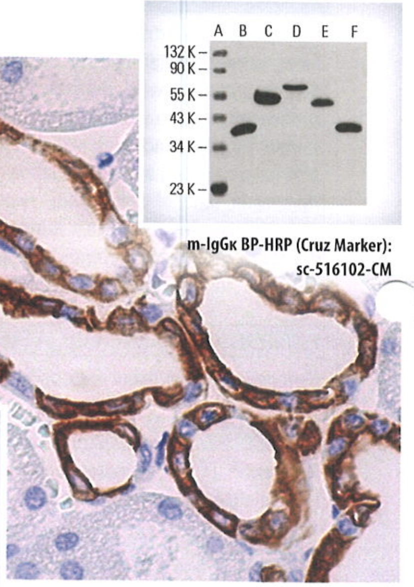

m-IgGκ BP-HRP (Cruz Marker): sc-516102-CM

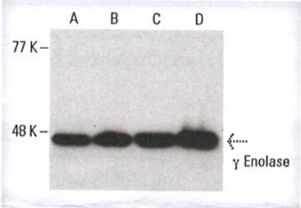

m-IgGκ BP-HRP: sc-516102 (WB & IHC)

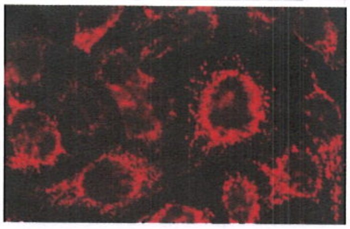

m-IgGκ BP-CFL 555: sc-516177

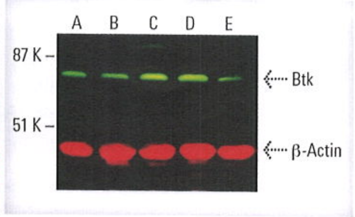

m-IgGλ BP-CFL 680: sc-516194
m-IgGκ BP-CFL 790: sc-516181

FREE MONOCLONAL ANTIBODY SAMPLES!

Contact our Technical Service Department (or local distributor) to identify the best monoclonal antibodies for your research needs. We will provide you with **trial size (10 µg) samples of monoclonal antibodies FREE of charge!**

Cruz Credit™ REWARDED

Write a review and receive 15 Cruz Credits™ per review

We receive valuable feedback from customers achieving better results with our monoclonal antibodies versus polyclonal antibodies. Share your monoclonal antibody results.

Find the product on our website and click the Write a Review link to submit your review.

www.scbt.com

SANTA CRUZ® BIOTECHNOLOGY
The Power to Question

abcam

Product datasheet

Anti-alpha smooth muscle Actin antibody ab5694

★★★★★ 138 Abreviews 1262 References 画像数 16

製品の概要

**製品名**	Anti-alpha smooth muscle Actin antibody
**製品の詳細**	Rabbit polyclonal to alpha smooth muscle Actin
**由来種**	Rabbit
**特異性**	Alpha smooth muscle actin antibody (ab5694) stains smooth muscle cells in vessel walls, gut wall, and myometrium. Myoepithelial cells in breast and salivary gland are also stained. ab5694 reacts with tumors arising from smooth muscles and myoepithelial cells. The other actins, such as beta- and gamma-cytoplasmic, striated muscle and myocardium are not stained by this alpha smooth muscle Actin antibody.
**アプリケーション**	**適用あり:** IHC-FoFr, ICC/IF, WB, ELISA, IHC-P, IHC-Fr
**種交差性**	**交差種:** Mouse, Rat, Chicken, Guinea pig, Cow, Dog, Human, Pig
**免疫原**	Synthetic peptide corresponding to Human alpha smooth muscle Actin. Alpha smooth muscle actin antibody (ab5694) was raised against a synthetic peptide corresponding to N-terminus of actin from human smooth muscle.<br>Database link: P62736
**ポジティブ・コントロール**	WB: HEK-293, NIH/3T3, Hela and jurkat whole cell lysate; Mouse heart tissue lysate; Rat2 myofibroblasts; Pig heart tissue lysate. ICC/IF: Pancreatic cancer cells. Rat myofibroblast cells. Human fetal heart cells IHC-P: Mouse intestine and mesentery tissue. Mouse mammary tissue
**特記事項**	Actins are highly conserved proteins expressed in all eucaryotic cells. Actin filaments form part of the cytoskeleton and play essential roles in regulating cell shape and movement. Six distinct actin isotypes have been identified in mammalian cells. Each is encoded by a separated gene and is expressed in a developmentally regulated and tissue-specific manner, alpha and beta cytoplasmic actins are expressed in a wide variety of cells; whereas, expression of alpha skeletal, alpha cardiac, alpha vascular and gamma enteric actins are more restricted to specialized muscle cell type. Smooth muscle alpha actin is of further interest because it is one of a few genes whose expression is relatively restricted to vascular smooth muscle cells. Further more, expression of smooth muscle alpha actin is regulated by hormones, cell proliferation and altered by pathological conditions including oncogenic transformation and atherosclerosis.

製品の特性

**製品の状態**	Liquid
**保存方法**	Shipped at 4°C. Store at +4°C short term (1-2 weeks). Upon delivery aliquot. Store at -20°C long term. Avoid freeze / thaw cycle.
**バッファー**	pH: 7.40<br>Preservative: 0.02% Sodium azide<br>Constituent: PBS
**精製度**	Immunogen affinity purified

**ポリ/モノ**	ポリクローナル
**アイソタイプ**	IgG

アプリケーション

Our Abpromise guarantee covers the use of **ab5694** in the following tested applications.

The application notes include recommended starting dilutions; optimal dilutions/concentrations should be determined by the end user.

アプリケーション	Abreviews	特記事項
IHC-FoFr		Use at an assay dependent concentration.
ICC/IF		1/100.
WB		Use a concentration of 0.5 - 2 µg/ml. Predicted molecular weight: 42 kDa.
E[illegible]A		Use a concentration of 0.1 - 1 µg/ml.
IHC-P		1/50 - 1/200. Perform heat mediated antigen retrieval before commencing with IHC staining protocol.
IHC-Fr		1/200. PubMed: 18559614Fix with acetone.

ターゲット情報

**機能**	Actins are highly conserved proteins that are involved in various types of cell motility and are ubiquitously expressed in all eukaryotic cells.
**関連疾患**	Defects in ACTA2 are the cause of aortic aneurysm familial thoracic type 6 (AAT6) [MIM:611788]. AATs are characterized by permanent dilation of the thoracic aorta usually due to degenerative changes in the aortic wall. They are primarily associated with a characteristic histologic appearance known as 'medial necrosis' or 'Erdheim cystic medial necrosis' in which there is degeneration and fragmentation of elastic fibers, loss of smooth muscle cells, and an accumulation of basophilic ground substance.
**配列類似性**	Belongs to the actin family.
**細胞内局在**	Cytoplasm > cytoskeleton.

画像

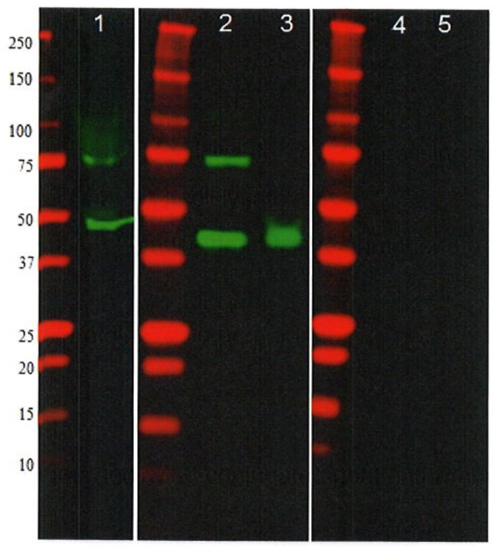

Western blot - Anti-alpha smooth muscle Actin antibody (ab5694)

Lanes 1-3 : Anti-alpha smooth muscle Actin antibody (ab5694) at 1 µg/ml

Lane 1 : HEK293 cell lysate - overexpressing alpha-Actin

Lanes 2 & 4 : 3T3 cell lysate

Lanes 3 & 5 : Mouse heart tissue homogenate

Lysates/proteins at 20 µg per lane.

Secondary

All lanes : Fluor 750-conjugated goat anti-rabbit IgG (H+L) at 1/12500 dilution

Predicted band size: 42 kDa

Observed band size: 42 kDa

Incubated with the primary antibody at 4°C overnight.

Incubated with the secondary antibody at room temperature for 1 hour.

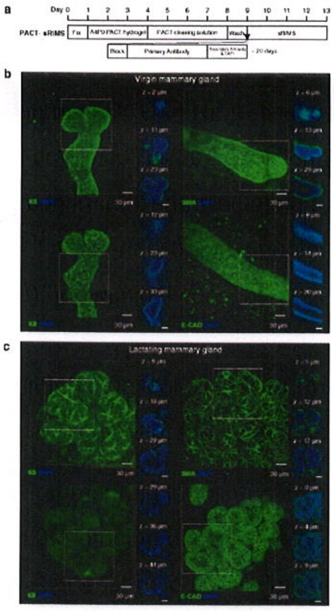

Immunohistochemistry (Formalin/PFA-fixed paraffin-embedded sections) - Anti-alpha smooth muscle Actin antibody (ab5694)

Image from Lloyd-Lewis B. et al., Breast Cancer Res. 2016 18: 127. Fig 2.; doi: 10.1186/s13058-016-0754-9. Reproduced under the Creative Commons license http://creativecommons.org/licenses/by/4.0/.

Passive CLARITY technique (*PACT*)-sorbitol refractive index matching solution (*sRIMS*) clearing and 3D imaging of virgin and lactating mouse mammary tissue. **a** PACT-sRIMS tissue clearing and immunostaining protocol and timeline. Three-dimensional confocal imaging of PACT-sRIMS-cleared virgin (**b**) and lactating (**c**) mammary glands immunostained with basal cell markers (K5 and smooth muscle actin (*stained with ab5694*)) and luminal cell markers (K8 and E-cadherin (*E-CAD*)). *Main image* shows the maximum intensity projection of the entire image sequence, with thin optical slices (1 µm) and their depth (*z* value) relative to the first image in the image sequence.

From a paper comparing imaging of intact virgin and lactating mammary glands using 3D imaging of solvent-cleared organs, see deep brain (seeDB), clear unobstructed brain imaging cocktails (CUBIC) and passive clarity technique.

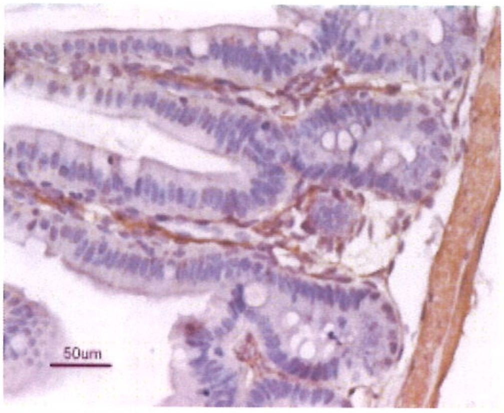

Immunohistochemistry (Formalin/PFA-fixed paraffin-embedded sections) - Anti-alpha smooth muscle Actin antibody (ab5694)

This picture shows formalin-fixed, paraffin embedded mouse intestine and mesentery, the optimal dilution is 1:1600 to 1:3200, incubation overnight at 4°C, counterstained with Hematoxylin.

This image was kindly supplied as part of the review by JQ Zhang.

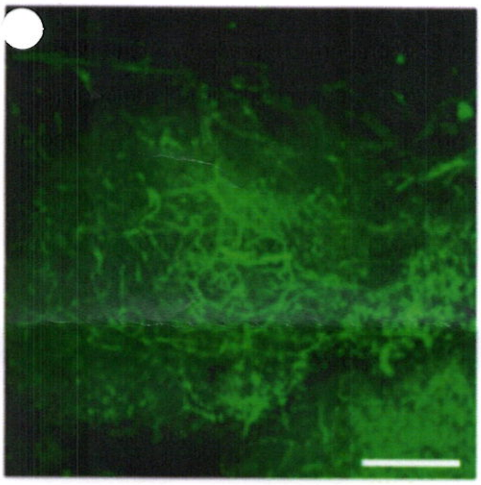

Immunohistochemistry (Formalin/PFA-fixed paraffin-embedded sections) - Anti-alpha smooth muscle Actin antibody (ab5694)

Image from Lee H. et al., BMC Dev Biol. 2014 Dec 21;14:48. Fig 2Bdoi: 10.1186/s12861-014-0048-3 Reproduced under the Creative Commons license http://creativecommons.org/licenses/by/4.0/.

Pancreatic vessel imaging in the intact adult mouse pancreas. In adult mouse tissues (12 weeks old), imaging was performed after CLARITY. Three-dimensional projection clarified mouse pancreas with capillary immunostained for α-smooth muscle actin (green). Scale bar, 200 μm.

From a study, that aimed to improve the original CLARITY procedure and developed specific CLARITY protocols for various intact organs.

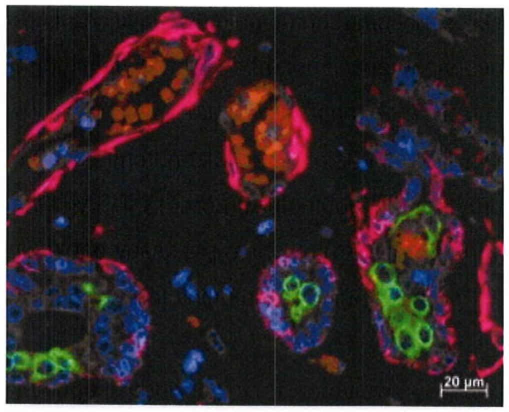

Immunohistochemistry (Frozen sections) - Anti-alpha smooth muscle Actin antibody (ab5694)

Ab5694 positively staining smooth muscle cells in blood vessels and myoepithelial cells in the frozen tissue of cancerous human mammary gland (pink) at 1/100 dilution. Secondary: CY5 conjugated goat anti rabbit (1/100). Co immunostaining of glandular cell cytokeratin can be seen stained by FITC (green). Auto fluorescent erythrocytes that are present within blood vessels are shown (red), whilst the DAPI counter stain may clearly be seen staining nuclei (blue).

This image is courtesy of an Abreview submitted by on **22 August 2005**. We do not have any further information relating to this image.

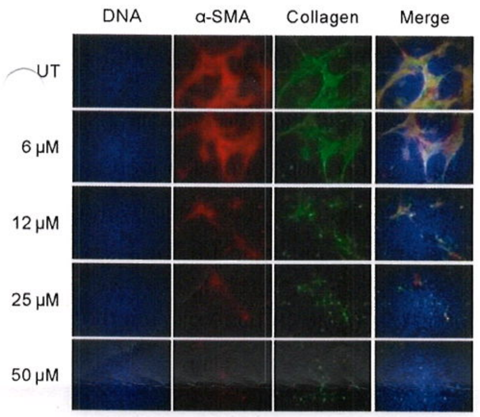

Immunocytochemistry/ Immunofluorescence - Anti-alpha smooth muscle Actin antibody (ab5694)

Watcott, C.J. et al PLoS One. 2017 Aug 25;12(8):e0183871. doi: [illegible]1371/journal.pone.0183871. eCollection [illegible]17 Reproduced under the Creative Commons license http://creativecommons.org/licenses/by/4.0/

Effects of the ROCK1 inhibition on pancreatic cancer cells and cancer-associated fibroblasts

Fluorescence microscopic analysis of fasudil treated, co-cultured pancreatic cancer cells and cancer-associated fibroblasts. Cells were treated with fasudil for 48 hours and then were stained for α-SMA (red), Collagen I (green), and DNA (blue).

Alpha smooth muscle Actin (α-SMA) is detected ising ab5694 in 5% formaldehyde-fixed cells.

(From Figure 3C of Watcott et al)

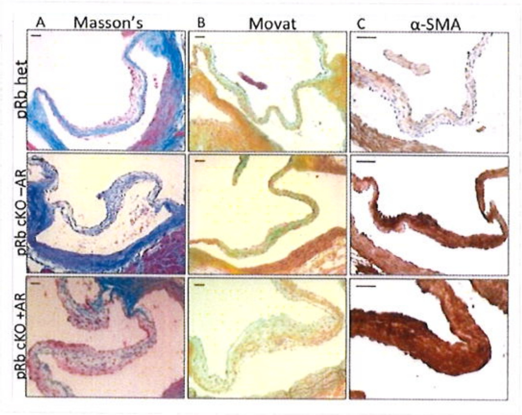

Immunohistochemistry (Formalin/PFA-fixed paraffin-embedded sections) - Anti-alpha smooth muscle Actin antibody (ab5694)

Freytsis, M. et al PLoS One. 2018 Jan 5;13 (1):e0190623. doi: 10.1371/journal.pone.0190623. eCollection 2018 Reproduced under the Creative [illegible]mmons license [illegible]p://creativecommons.org/licenses/by/4.0/

Representative histology of aortic valve leaflets from aged mice demonstrates changes in pRb cKO AoV

A) Masson's trichrome showing reduced collagen staining (blue) in leaflet from pRb cKO mouse with aortic regurgitation (AR). B) Movat pentachrome showing more diffuse collage staining (yellow) in fibrosa, but normal proteoglycan staining (blue) in the spongiosa layer of the leaflet from pRb cKO with AR. **C) Immunohistochemistry for α-SMA, demonstrating presence of activated myofibroblasts throughout leaflets of pRb cKO mouse with and without AR.** Scale bar is 50μm.

Alpha smooth muscle Actin is detected witn ab5694 at 1/1000 dilution.

(From Figure 2 of Freytsis et al)

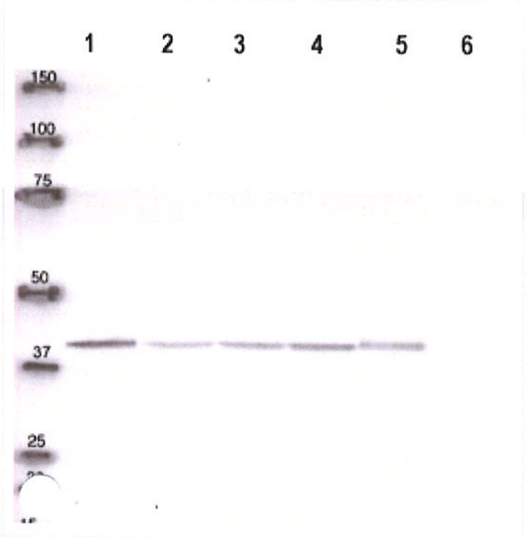

Western blot - Anti-alpha smooth muscle Actin antibody (ab5694)

All lanes : Anti-alpha smooth muscle Actin antibody (ab5694) at 1/500 dilution

Lane 1 : Rat2 myofibroblasts (untreated before treatment-0 days)
Lane 2 : Rat2 myofibroblasts (untreated for 5 days)
Lane 3 : Rat2 myofibroblasts (treated with 1ng/mL TGF beta)
Lane 4 : Rat2 myofibroblasts (treated with 10ng/mL TGF beta)
Lane 5 : Positive control (NIH3T3)
Lane 6 : Negative control (MDA-MB-469 breast carcinoma cells)

Lysates/proteins at 10 μg per lane.

Secondary
All lanes : Donkey anti rabbit (HRP) at 1/2500 dilution

Performed under reducing conditions.

Predicted band size: 42 kDa

This image is an edited version of an image submitted courtesy of an Abreview on **20 September 2005**. We do not have any further information relating to this image.

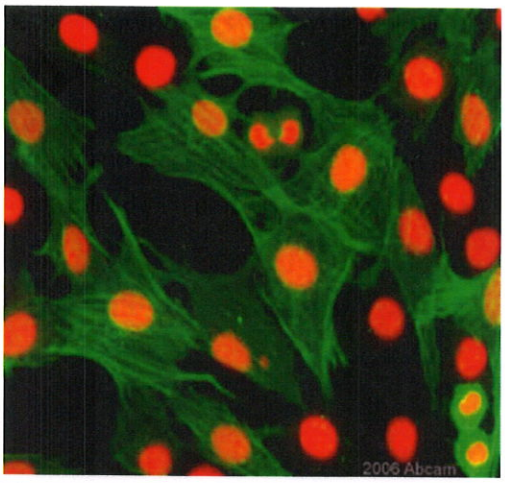

Immunocytochemistry/ Immunofluorescence - Anti-alpha smooth muscle Actin antibody (ab5694)

This image is courtesy of an Abreview submitted by Dr Jianyuan Chai

ab5694 at 1/500 staining rat myofibroblast cells by Immunocytochemistry/ Immunofluorescence. The cells were formaldehyde fixed and blocked with 5% serum prior to incubation with the antibody for 2 hours. A FITC conjugated goat anti-rabbit IgG was used as the secondary. Nuclei were counterstained with propidium iodide.

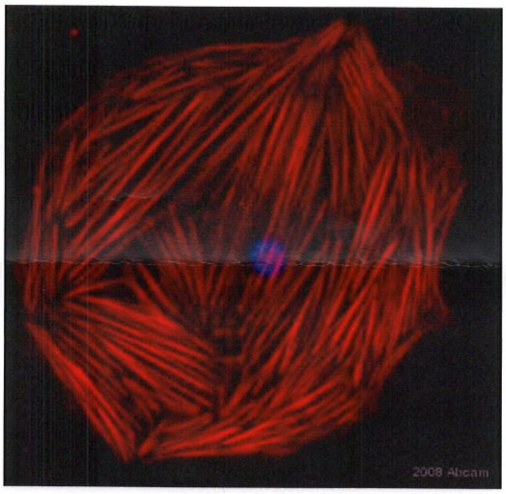

Immunocytochemistry/ Immunofluorescence - Anti-alpha smooth muscle Actin antibody (ab5694)

This image is courtesy of an anonymous Abreview

ab5694 staining Human fetal heart cells by ICC/IF. Cells were fixed with PFA and permeabilized in 0.1% saponin prior to blocking in 10% serum for 45 minutes at 37°C. The primary antibody was diluted 1/400 and incubated with the sample for 1 hour at 37°C. A Alexa Fluor® 594 conjugated goat polyclonal to rabbit IgG (H+L), diluted 1/600 was used as secondary antibody.

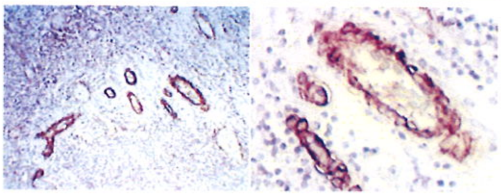

Immunohistochemistry (Formalin/PFA-fixed paraffin-embedded sections) - Anti-alpha smooth muscle Actin antibody (ab5694)

Immunohistochemistry (Formalin/PFA-fixed paraffin-embedded sections) analysis of human tonsil tissue labelling alpha smooth muscle Actin with ab5694 at a dilution of 1/1000. Heat mediated antigen retrieval was performed for 35 minutes followed by cooling for 20 minutes. Sections were incubated with the primary antibody for 1 hour followed by incubation with a biotinylated secondary antibody for 30 minutes then HRP-Streptavidin for 30 minutes. Developed using DAB chromogen substrate (5-10 minutes). Counter stained with hematoxylin.

Magnification: left - 10X, right - 40X.

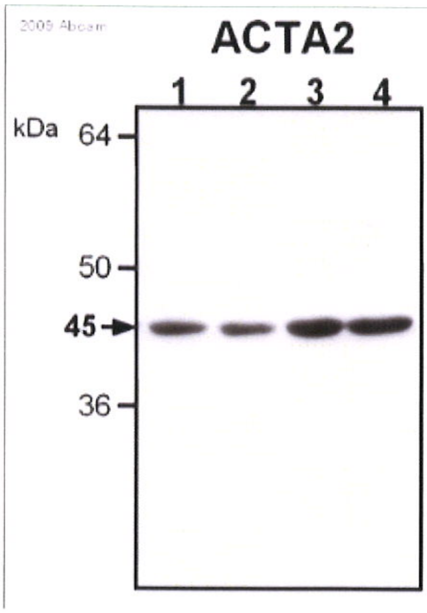

Western blot - Anti-alpha smooth muscle Actin antibody (ab5694)

s image is a courtesy of Mario Torrado

All lanes : Anti-alpha smooth muscle Actin antibody (ab5694) at 1/1000 dilution

Lanes 1-2 : Lystates prepared from pig heart tissue from normal control animals

Lanes 3-4 : Lystates prepared from pig heart tissue from experimental animals

Lysates/proteins at 4 µg per lane.

Secondary

All lanes : HRP-conjugated goat polyclonal to rabbit IgG at 1/20000 dilution

Performed under reducing conditions.

Predicted band size: 42 kDa

Observed band size: 45 kDa

why is the actual band size different from the predicted?

Exposure time: 1 minute

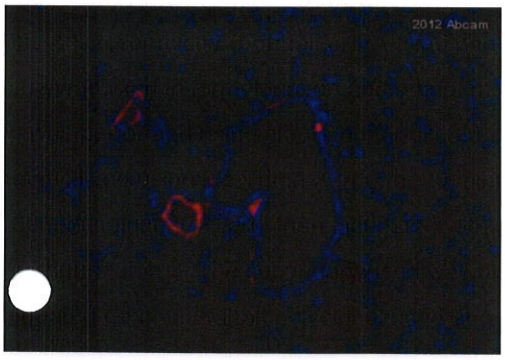

Immunohistochemistry (Formalin/PFA-fixed paraffin-embedded sections) - Anti-alpha smooth muscle Actin antibody (ab5694)

Image courtesy of an anonymous Abreview.

ab5694 staining alpha smooth muscle Actin in rat lung tissue by Immunohistochemistry (Formalin/PFA-fixed paraffin-embedded sections). Tissue was fixed in formaldehyde and a heat mediated antigen retrieval step was performed using citrate buffer pH 6.0. Samples were then permeabilized using 0.5 Triton X-100 for 20 minutes, blocked with 1% BSA for 30 minutes at 20°C and then incubated with ab5694 at a 1/100 dilution for 16 hours at 4°C. The secondary used was an Alexa-Fluor 568 conjugated goat anti-rabbit polyclonal used at a 1/250 dilution.

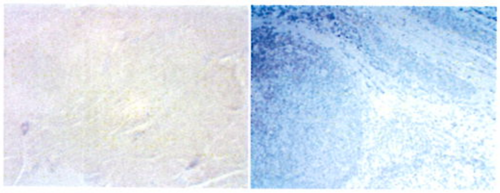

Immunohistochemistry (Formalin/PFA-fixed paraffin-embedded sections) - Anti-alpha smooth muscle Actin antibody (ab5694)

Immunohistochemistry (Formalin-fixed paraffin-embedded sections) analysis of skeletal muscle tissue (left) incubated with ab5694 at 1/100 at room temperature for 1 hour showing no specific staining. Right - human tonsil tissue secondary only control.

Heat mediated antigen retrieval was performed for 35 minutes followed by cooling for 20 minutes. A biotinylated secondary antibody was used for 30 minutes followed by incubation with HRP-Streptavidin for 30 minutes. Developed using DAB chromogen substrate (5-10 minutes). Counter stained with hematoxylin. Magnification 10X.

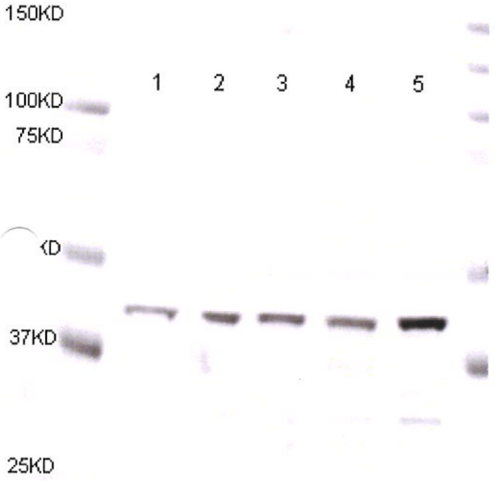

Western blot - Anti-alpha smooth muscle Actin antibody (ab5694)

All lanes : Anti-alpha smooth muscle Actin antibody (ab5694) at 1 µg/ml

Lane 1 : HeLa Nuclear
Lane 2 : HeLa whole cell
Lane 3 : A431 cell lysate
Lane 4 : Jurkat cell lysate
Lane 5 : HEK293 cell lysate

Lysates/proteins at 20 µg per lane.

Secondary
All lanes : Alexa Fluor anti-rabbit at 1/5000 dilution

Performed under reducing conditions.

Predicted band size: 42 kDa
Observed band size: 42 kDa
Additional bands at: 30 kDa, 35 kDa, 37 kDa, 50 kDa, 75 kDa. We are unsure as to the identity of these extra bands.

Please note that ab5694 does not appear to be specific to smooth muscle.

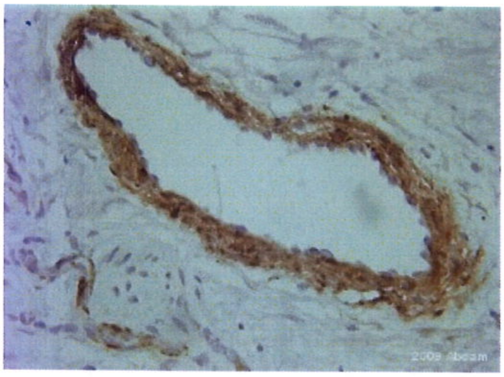

ab5694 staining alpha smooth muscle Actin in human skin tissue section by Immunohistochemistry (Formalin/PFA-fixed paraffin-embedded sections). Tissue underwent formaldehyde fixation before heat mediated antigen retrieval in Citrate pH 6.0 and then blocked with 10% serum for 1 hour at RT. The primary antibody was diluted 1/300 and incubated with sample in 2% serum for 15 hours at 4°C. A Biotin conjugated goat polyclonal to rabbit IgG was used at dilution at 1/500 as secondary antibody.

Immunohistochemistry (Formalin/PFA-fixed paraffin-embedded sections) - Anti-alpha smooth muscle Actin antibody (ab5694)

This image is courtesy of an Anonymous Abreview

Please note: All products are "FOR RESEARCH USE ONLY. NOT FOR USE IN DIAGNOSTIC PROCEDURES"

Our Abpromise to you: Quality guaranteed and expert technical support

- Replacement or refund for products not performing as stated on the datasheet
- Valid for 12 months from date of delivery
- Response to your inquiry within 24 hours
- We provide support in Chinese, English, French, German, Japanese and Spanish
- Extensive multi-media technical resources to help you
- We investigate all quality concerns to ensure our products perform to the highest standards

If the product does not perform as described on this datasheet, we will offer a refund or replacement. For full details of the Abpromise, please visit https://www.abcam.co.jp/abpromise or contact our technical team.

Terms and conditions

- Guarantee only valid for products bought direct from Abcam or one of our authorized distributors
